# Exploring the Effects of Tasks with Different Decision-Making Levels on Ball Control, Passing Performance, and External Load in Youth Football

**DOI:** 10.3390/children10020220

**Published:** 2023-01-26

**Authors:** Diogo Coutinho, Adam Leigh Kelly, Sara Santos, Pedro Figueiredo, David Pizarro, Bruno Travassos

**Affiliations:** 1 University of Maia (UMAIA), 4475-690 Maia, Portugal; 2 Department of Sports Sciences, Exercise and Health, University of Trás-os-Montes and Alto Douro, 5000-801 Vila Real, Portugal; 3 Research Center in Sports Sciences, Health Sciences and Human Development (Centro de Investigação em Desporto, Saúde e Desenvolvimento Humano, CIDESD), 5000-801 Vila Real, Portugal; 4 Research Centre for Life and Sport Sciences (CLaSS), Birmingham City University, Birmingham B15 3TN, UK; 5 Physical Education Department, College of Education, United Arab Emirates University, Al Ain P.O. Box 15551, United Arab Emirates; 6 Portugal Football School, Portuguese Football Federation, 4711-852 Oeiras, Portugal; 7 Centro de Estudios Superiores Don Bosco (CES Don Bosco), Universidad Complutense de Madrid (UCM), 28040 Madrid, Spain; 8 Faculty of Life Sciences and Nature, University of Nebrija, 28015 Madrid, Spain; 9 Department of Sports Sciences, University of Beira Interior, 6201-001 Covilhã, Portugal

**Keywords:** perception–action, training tasks, technical performance, ball control, passing behaviour, team sports

## Abstract

This study aimed to understand how the design of decision-making tasks affects youth football players’ ball control, passing performance, and external load. A total of 16 male youth football players (age: 12.94 ± 0.25 years) competed in various tasks based on the following levels of decision-making: (i) low decision-making (Low DM), which consisted of a predefined ball control and passing sequence; (ii) moderate decision-making (Mod DM), which consisted of maintaining possession in a square with four players and two balls while maintaining the same position; and (iii) high decision-making (High DM), which consisted of a 3 vs. 3 + 2 neutral players ball possession game. The study design consisted of a pre–post design (a 6 min pre-test game, a 6 min intervention, and a 6 min post-test game). The players’ ball control and passing performance were measured using the game performance evaluation tool and notational analysis, while GPS data were used to determine their physical performance. The pre–post test analysis revealed decrements in players’ ability to identify more offensive players after the Mod DM task (W = 9.50, *p* = 0.016), while there was an increase in their ability to receive the ball towards the space following the High DM task (t = −2.40, *p* = 0.016). Analysis between groups showed lower values in most ball control variables for the Low DM task compared to the Mod DM task (ball control execution, *p* = 0.030; appropriateness, *p* = 0.031; motor space, *p* = 0.025), while there were also lower values in the distance covered while sprinting (*p* = 0.042). Overall, prescriptive tasks (Low DM) that are repetitive in nature may affect players’ perceptual attunement, whereas static tasks (e.g., Mod DM) may limit their ability to locate players in more offensive positions. Moreover, game-based situations (High DM) seem to acutely enhance players’ performance, possibly due to contextual dependency. Overall, coaches should carefully consider the type of practice structure when designing tasks that aim to improve players’ technical skills in youth football.

## 1. Introduction

Association football is a sport where two teams compete dynamically in space and time to unfold goal-direction behaviours [1,2]. To this end, players adjust their positioning and behaviour according to the spatiotemporal information that they perceive, such as the distance and angle between teammates and opponents [2,3]. In this view, opportunities for action (i.e., *affordances*) constantly change according to variations in the context of play or in the player’s exploration of the environment [4,5]. Thus, successful performance in team sports appears to depend on players’ positioning to perceive and act. This emphasises the importance of decision-making (DM) processes in order to succeed within specific competitive environments. 

To improve players’ skills, a wide body of research has explored how different training approaches impact players’ development. Traditionally, training approaches in team sports have adopted analytical tasks by prescribing specific movement patterns that reduce DM, as players’ actions are often predetermined [6,7]. For example, from a technical perspective, players are often exposed to repetitive blocked practices (e.g., groups of two players passing the ball to one another in a static position), which decrease attentional demands [8,9]. These activities are usually performed during earlier phases of the training session and/or learning phase [8,10,11,12], following which the players are exposed to game-based scenarios where it is expected that such skills are transferred [13,14,15]. However, this approach has received criticism because it decouples perception from action [8], preventing individuals from perceiving when or how to use such skills [6,13].

More recently, small-sided games have been suggested as an appropriate and relevant training tool, as they allow for concomitant development of players’ technical, physical, and tactical aspects of play [16,17]. In addition, coaches can vary the boundary conditions during these game-based scenarios to emphasise specific behaviours [18]. For example, coaches may reduce the size of the pitch to increase the frequency of ball control [19] and passing actions [20], or they may even limit the number of touches to encourage passing behaviour [21]. However, most studies that have explored game-based situations focus on the frequency of actions, without considering DM [22,23]. Developing a better understanding of the effects that different tasks may have on such skills may help coaches to tailor more appropriate training interventions. 

When considering a player’s development, mainly at younger ages (i.e., under-7 (U7) to U14), one major focus is on developing their technical capabilities [24,25]. Based on these technical skills, the ability to control [26,27] and pass the ball [28,29] are among the most relevant to be successful and attain higher performance levels [26,30,31,32]. Previous results exploring the DM and execution of passing behaviour showed that older players (U12/14) revealed higher values when compared to their younger counterparts (U8/U10) [33]. First, these findings suggest that players within the U12/14 category have a higher tactical awareness that allows them to locate relevant solutions within a competitive environment; second, coaches should design training practices that require such skills. In this respect, several studies have explored different training strategies to develop players’ DM and execution of ball control or passing skills [34,35,36,37]. For example, Práxedes, Moreno, Gil-Arias, Claver, and Del Villar [36] displayed how the DM and pass execution of U12 football players improved after the practice of a numerical superiority exercise.

Despite the growing contribution of research to understanding how different training interventions affect youth players’ ball control and passing performance [34,35,36,38], less scientific information is available in relation to the acute effects that different types of training tasks (i.e., more prescriptive or game-based) may have. For instance, despite game-based situations having been suggested as practices that may be more relevant to prepare players to act during competitive performances, coaches of youth players still spend a vast amount of time using prescriptive and repetitive tasks [39]. Therefore, it is important to explore the role of such training tasks to better understand players’ motor execution and DM [40]. Thus, this study aimed to examine how the manipulation of contextual dependency and DM affects players’ positioning and subsequent ball control, passing performance, and external load during SSGs. It was hypothesised that tasks with low-to-moderate DM would ensure low ball control and passing performance transfer to subsequent competitive tasks, due to the lower contextual dependency and DM. Additionally, it was expected that training tasks that required lower DM would lead to higher decrements in players’ performance compared to tasks that required higher levels of DM. Lastly, it was expected that the type of training task adopted would lead to different effects on the players’ ability to control or pass the ball, as well as on their physical performance. 

## 2. Materials and Methods

### 2.1. Participants

A total of 20 youth male football players (age = 12.94 ± 0.25 years; height = 155.75 ± 6.24 cm; weight = 45.13 ± 9.62 kg; football experience = 5.56 ± 1.9 years) from a Portuguese club competing at the regional level volunteered to participate in this study. All players belonged to the same club, engaged in three training sessions per week (~90 min per training session), and played an official 11-a-side match during the weekend. Four goalkeepers participated in the study; however, considering their specific positioning on the pitch (i.e., a more regular and static positioning), their data were excluded from the data analysis. The participants included additional players who were subsequently excluded as a result of (i) injury or illness prior to the data collection (n = 1) and (ii) reporting an intention to not be present at one of the data collection sessions (n = 3). Informed and written consent was provided by the club, the head coach, the players, and their legal guardians before the start of the data collection. The study protocol adhered to the guidelines of the ethics committee of the local university and the recommendations of the Declaration of Helsinki.

### 2.2. Procedures 

This study explored the acute effects of exposing the players to different positioning and passing tasks. For this purpose, players were exposed to a pre–post test design. Accordingly, the players performed one SSG bout followed by one of three possible experimental intervention tasks: (i) low DM (Low DM), (ii) moderate DM (Mod DM), and (iii) high DM (High DM). The players then performed one additional SSG bout under the same conditions to understand how these training tasks acutely modified the players’ ball control, passing performance, and external load. The players were tested in a total of seven sessions on non-consecutive days (i.e., across four weeks on their 18:30–20:00 h Monday and Thursday sessions) during the competitive period (mid-season; November–December from the 2022–2023 season). The first session was developed for familiarisation purposes. 

**Figure 1:**
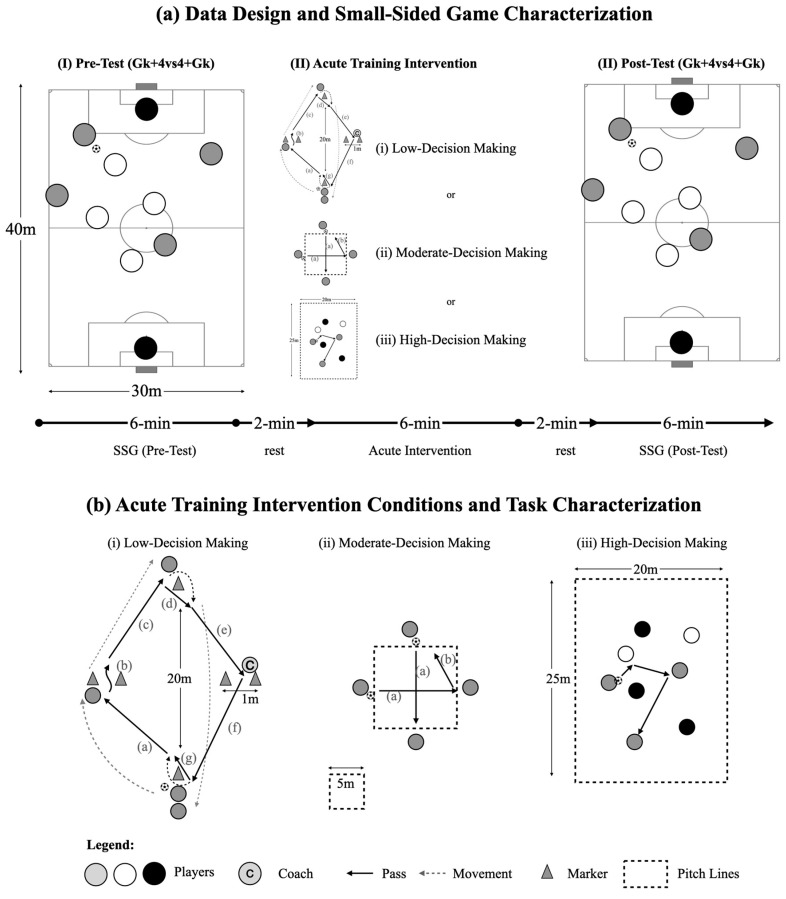
Representation of data design and acute training interventions.

On all days, the sessions began with a standardised 15 min warm-up consisting of mobility-based movements and a possession game (4 vs. 4 without goals). Following the warm-up, the players were allocated to one of the pitches (Pitch 1, Team A vs. Team B; Pitch 2, Team C vs. Team D) to perform a Gk + 4 vs. 4 + Gk SSG using official 7-a-side goals on a 40 × 30 m artificial turf pitch (length × width ratio = 1.33). The game lasted for 4 min and was used to assess the players’ performance prior to the training intervention (pre-test). Several official-sized footballs were placed near the pitch’s external lines to guarantee the ball’s fast replacement, decreasing the time spent out of play. No coach feedback or encouragement was allowed during the pre- and post-test SSGs (i.e., during the game situations used to measure the players’ performance, so as not to affect the players’ performance). The SSG was performed according to the official FIFA rules on an outdoor artificial turf pitch, with the following exceptions: (i) the game restarted by the corresponding goalkeeper when a goal was scored or when the ball left the pitch by the end line, allowing a faster restart; and (ii) no offside rule was applied. After the pre-test, the players had a 2 min rest period, in which they were encouraged to drink water, followed by the 6 min intervention tasks (Low, Mod, and High DM) in a random order set using random.org. Lastly, the SSG was repeated 2 min after the intervention to inspect how the intervention acutely impacted the players’ performance (post-test) (Figure 1a). 

### 2.3. Training Intervention

For the purpose of the study, the players were exposed to three experimental conditions (see Figure 1). First, during the Low DM conditions, the players performed a prescribed drill focused on positioning, the orientation of ball reception that is, oriented ball reception at first touch between the markers and then pass the ball towards the following teammate.. Players were constantly instructed to maintain the passing rhythm and correctly perform a high number of passes using the foot to open space to progress between the markers (Figure 1b, left panel). Second, during the Mod DM conditions, each player was required to adjust their positioning, the orientation of ball reception, and their passing to maintain the possession of two balls simultaneously [41] in a square of 5 × 5 m. Players were constantly encouraged by the head researcher (i.e., UEFA A holder with more than 15 years of training experience) to orient their bodies according to the ball’s location, to receive the ball oriented, and to always pass according to the other ball’s location, avoiding one player having two balls (Figure 1b, middle panel). Lastly, during the High DM conditions, the players performed a ball possession game consisting of a 3 vs. 3 + 2 neutral players on a 25 × 20 m pitch [20], while also playing two mandatory touches to stress body orientation, ball control, and passing ability [21]. The players were constantly encouraged to orient their bodies to view all teammates, to receive the ball oriented to open space, and to pass the ball to the free teammate and ensure ball possession (Figure 1b, right panel).

Before these tasks, the lead researcher (first author) provided feedback to the players regarding orientation, ball control, and passing action. In this respect, for the body orientation, the players were always encouraged to see all of their teammates and to receive the ball with their body oriented to the following passing direction and the opponent’s target. For the ball control, players were encouraged in terms of (i) the ability to receive the ball with the body surface in such a way as to guarantee better conditions in terms of space and time to decide (e.g., using the right foot while playing in the right wide channel, rather than the left foot, which may limit passing opportunities), and (ii) having the ball in the motor space, which relates to the ability to retain possession by controlling the body’s stiffness (e.g., being able to cushion the ball when receiving a hard ball, or to push the ball forward if it comes soft). In contrast, for the passing behaviour, the players were encouraged in terms of (i) the importance of considering how far forward the free teammate is from the closest defender (e.g., exploring passes to the wide channel to move the defenders so as to further explore the centre channel); (ii) offensive solutions, including being able to identify a player that may allow progress towards the opposing target, or to destabilise the opposing team’s defensive behaviour and compactness (e.g., passing the ball to the centre channel to attract opposition to further perform a pass towards a free teammate); and (iii) rhythm and tempo, which relates to being able to adjust the passing length, speed, and direction according to the teammates’ and opponents’ movements, mainly by reinforcing the pass towards the front of the free teammate. 

### 2.4. Data Collection 

#### 2.4.1. Ball Control, Passing Execution, and Decision-Making (GPET)

The SSGs were recorded using two digital video cameras (Panasonic NV-GS230) positioned at a height of 2 m and aligned with the central section of the pitch. The LongoMatch software, version 1.3.7 (LongoMatch, Fluendo, Barcelona, Spain), was used for the notational analysis of the players’ ball control and passing performance. 

**Figure 2:**
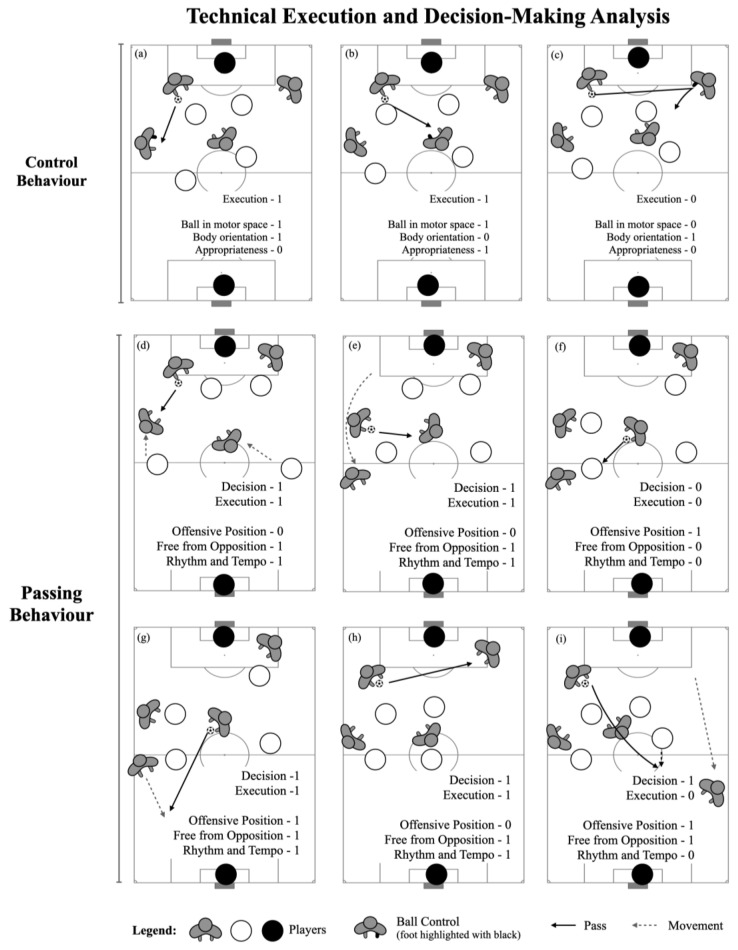
Representation of technical criteria of ball control and passing patterns. (**a**–**c**) analyses players’ ball control according to GPET instrument and the additional technical criteria to highlight how each criteria should be coded according to the specific context. (**d**–**i**) have also GPET and technical criteria analysis however, regarding the players’ passing behaviour. Note: dashed line represents players’ movement, while continuous line refers to ball trajectory.

The players’ execution and decision-making ability were measured using the game performance evaluation tool (GPET) [42]. This tool has been used to measure players’ DM and execution during youth soccer SSGs [36,42,43]. The execution of ball control was coded as 0 if the player was not able to properly control the ball within their motor space (e.g., ball bouncing to another teammate or opposing player), while it was coded as 1 if they were able to control it to play further (i.e., pass, travel with the ball, dribble, or shoot) [42]. Regarding the pass, the decision-making was coded as 0 if the pass was performed to a teammate closely marked by an opponent or executed to an area of a pitch without any teammate (see Figure 2f), while a value of 1 was awarded if the pass was performed to a free teammate (see Figure 2e,g). The motor execution of the pass was coded as 0 if the pass did not reach the target player (see Figure 2i), while it was coded as 1 if the pass reached the target teammate (see Figure 2d,e for reference). Both the DM (of the passing) and motor execution (from ball control and passing behaviour) were then presented as the percentage of successful related decisions over the total number of actions performed (e.g., the motor execution for the pass was coded as: successful passes / (successful passes + unsuccessful passes)) [44]. A total of 918 actions were recorded (ball control motor execution n = 450; passing behaviour motor n = 468). All videos were coded by the same expert analyst, with more than 10 years of experience in training and match analysis. The intra-observer correlation was developed considering 10% of the sample. The values ranged from 0.83 to 0.94 for the different categories, which were deemed high and within the thresholds presented in previous reports [42]. 

#### 2.4.2. Technical Criteria of Ball Control and Passing 

The same procedures were used to code the technical criteria of the players’ ball control and passing actions. The following criteria were used to code the players’ ball control (see Figure 2): (i) players’ ability to receive the ball with the relevant body part to control the ball (see Figure 2a); (ii) players’ ability to maintain the ball in the motor space (see Figure 2b); and (iii) players’ body orientation and ability to receive the ball with the intention to progress towards the opponents’ goal (see Figure 2c). 

From the passing behaviour, the following technical criteria were used: (i) free from opposition, which is the player’s ability to pass the ball to a teammate who is not closely marked by the opposition (e.g., see Figure 2g for a proper passing situation, and Figure 2f for a pass to a marked teammate); (ii) offensive position, which is the players’ ability to perform passes that allow progress on the field (for example, Figure 2h reflects a situation in which there were more offensive solutions than the selected pass); and (iii) rhythm and tempo, which reflect the players’ ability to pass the ball with proper speed and direction (see Figure 2i,h for examples). These criteria were created to complement the information from the GPET measures, which decompose the skills into more specific and contextual information. Following the data collection and one week later, 10% of the sample was retested, and the values varied from 0.89 to 0.84 for both the ball control and passing behaviours (i.e., high intraclass correlation) [45].

#### 2.4.3. Physical Performance

Physical data during the SSGs were gathered using 10 Hz Global Positioning System (GPS) units (10 Hz, Accelerometer 1 kHz, FieldWiz, Paudex, Switzerland). These devices were placed in a specific vest on the upper backs of the players, who always used the same GPS device to reduce error. The FieldWiz GPS trackers have been shown to have a good level of accuracy for measuring movements and displacements in team sports [46]. In this respect, the total distance covered and the distance covered by the players in different speed zones were in accordance with the following thresholds adopted by previous studies analysing SSGs with youth players [21,47]: (i) total distance covered; (ii) distance covered while walking (0.0–3.5 km/h); (iii) distance covered while jogging (3.6–14.3 km/h); (iv) distance covered while running (14.4−19.8 km/h); and (v) distance covered while sprinting (>19.9 km/h). In addition, the players’ average speed (m/s) was considered to understand the game’s pace.

### 2.5. Statistical Analysis

Descriptive data were presented as the mean (M) and standard deviation (SD) for data following a normal distribution, and as the median (Me) and interquartile range (IQR) for data showing non-normal distribution. Evaluation for outliers and assumptions of normality were tested using the Shapiro–Wilk test. The differences between the pre- and post-test measures of each condition (i.e., within comparisons for Low DM, Mod DM, and High DM) were analysed using Student’s *t*-test for variables with a normal distribution, while the Wilcoxon test was used for variables with non-normal distribution. Statistical significance was set at *p* < 0.05, and calculations were performed using the Jamovi Project (Computer Software Version 1.2. 2020). To examine the differences in means, 95% confidence limits (raw data) and Cohen’s d effect sizes were applied to the pairwise comparisons. The thresholds for effect size statistics were as follows: 0.0–0.19 (trivial); 0.20–0.49 (small); 0.6–1.19 (moderate); 1.2–1.9 (large); ≥ 2.0 (very large) [48].

As a result of the inequality in the pre-test values for the technical variables, analysis of covariance (ANCOVA) was used to compare the different training tasks in the intervention, with post-test values as the dependent variable and pre-test values as the covariate. For each ANCOVA, the differences between training tasks were measured using the partial eta-squared (η^2^), which was calculated using the following thresholds: 0.01 (small), 0.06 (medium), and 0.14 (large) [49]. 

## 3. Results

### 3.1. Ball Control and Passing Actions during the Intervention Tasks

Descriptive results from the frequency of ball control and passing behaviour (i.e., successful and unsuccessful motor executions) are outlined in Table 1. These variables are expressed as the frequency per minute for purposes of better comparison. The comparison between the intervention tasks shows higher numbers of ball control and passing actions in Mod DM compared with the Low and High DM tasks (Table 1). 

### 3.2. Effects of the Intervention Tasks on Players’ Ball Control and Passing Actions (Within-Group Analysis)

**Figure 3:**
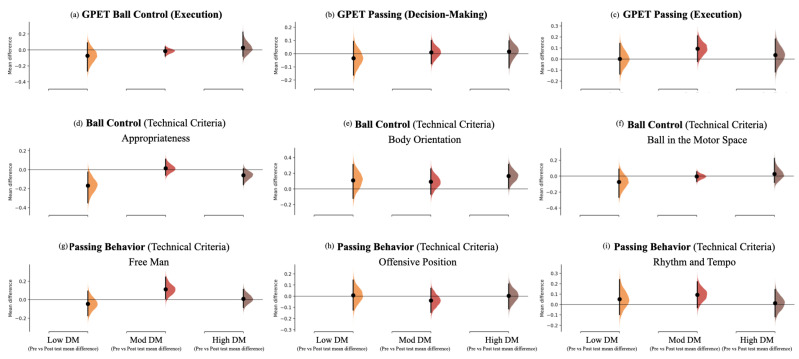
The mean difference (each mean difference is plotted as a bootstrap sampling distribution) for three comparisons pre- and post-measurements according to (i) GPET ball control, (ii) GPET passing execution, and (iii) GPET DM for the Low DM, Mod DM, and High DM tasks, as shown in the Cumming estimation plots above. The raw data are plotted on the upper axes. Mean differences are depicted as dots; 95% CIs are indicated by the ends of the vertical error bars [50].

**Figure 4:**
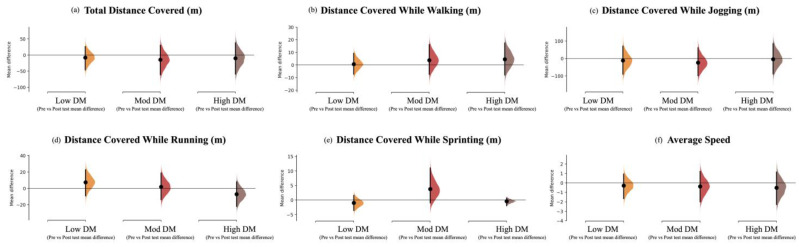
The mean difference (each mean difference is plotted as a bootstrap sampling distribution) for three comparisons pre- and post-measurements according to (i) total distance covered, (ii) distance covered while walking, (iii) distance covered while jogging, (iv) distance covered while running, (v) distance covered while sprinting, and (vi) average speed for the Low DM, Mod DM, and High DM tasks, as shown in the Cumming estimation plots above. Mean differences are depicted as dots; 95% CIs are indicated by the ends of the vertical error bars [50].

The acute effects of each task on the players’ subsequent performance (i.e., comparing the pre-test and post-test results for each condition) are presented in Table 2 (identified by the # signal) and Figure 3 and Figure 4. The High DM task contributed to acute improvements in body orientation (t = −2.40, *p* = 0.016; ES with 95% CI: ES = 0.67 [0.07; 1.27]). In contrast, there were decrements in identifying teammates in more offensive positions after the Mod DM intervention (−0.04; ±0.10 lower; W = 9.50, *p* = 0.016; ES = −0.22 [−0.80; 0.37]). 

Mean changes are depicted in Figure 3 and Figure 4. For example, from the GPET perspective (Figure 3a–c), the High DM task revealed improvements in the mean values for all variables, while the Mod DM task only showed improvements for passing behaviour, and the Low DM task revealed decrements for ball control and passing decision-making. 

### 3.3. Comparing the Interventions’ Acute Effects between Groups (Between-Group Analysis)

The general comparison between groups (i.e., Low DM, Mod DM, and High DM) showed effects for the ball control execution (F = 3.71, *p* = 0.032), appropriateness (F= 4.49, *p* = 0.017), motor space (F = 3.89, *p* = 0.028), and distance covered while sprinting (F = 3.42, *p* = 0.042). Accordingly, lower values of ball control execution (*p* = 0.030, −0.99 [−1.79; −0.21]), appropriateness (*p* = 0.031, −0.93 [−1.69; −0.19]), motor space (*p* = 0.025, −0.93 [−1.81; −0.23]), and distance covered while sprinting (*p* = 0.05, −0.84 [−1.58; −0.11]) were found after the Low DM task compared to the Mod DM task. Furthermore, the Low DM task revealed a general decrease in performance in ball control appropriateness (*p* = 0.037, 0.92 [0.17; 1.66]) compared to the High DM task.

## 4. Discussion

This study aimed to understand how the design of contextual dependency and DM of training tasks affect players’ positioning and, consequently, their ball control, passing performance, and external load during a subsequent performance in SSGs. Overall, the results identified several possible acute effects on the players’ subsequent game performance due to varying the tasks’ DM levels. More prescriptive tasks (i.e., Low DM and Mod DM) contributed to a higher frequency of actions but, as expected, to a lower transfer to the subsequent game performance because of the lower contextual dependency. In turn, adopting tasks such as the game (i.e., High DM task) appeared to emphasise the coupling between perception and action, thereby enhancing the players’ acute response. This evidence may support the improvements in receiving with the ball oriented following the High DM task, as well as the general improvements in DM and execution of both ball control and passing behaviour. In addition, following the Mod DM task, there was a reduction in the players’ ability to find more offensive solutions, in agreement with our hypothesis, which suggests that the configuration of each training task will impact the players’ performance in the subsequent task. Decrements in ball-control-related variables were also identified after the Low DM task, mainly when compared to the Mod DM task, which may have resulted from the lower perceptual demands of this type of practice. 

### 4.1. Effects of the Intervention Tasks on Players’ Ball Control and Passing Actions (Within-Group Analysis)

During training sessions, coaches often plan tasks under one or two specific topics (e.g., changing the point of attack, developing finishing behaviours), in which there is a progressive structure in terms of contextual dependency and decision-making process [6,51]. For example, before a task intended to develop the offensive process (e.g., 7 vs. 4 + Gk), coaches usually perform more repetitive tasks (e.g., specific set of players in 11 vs. 0 + Gk) that highlight the individual/collective possibilities for actions that need to be performed to be successful in the context of performance. It is considered that the low difficulty, complexity, and variability of such tasks will contribute to a high frequency of actions, subsequently promoting a better transfer to competitive scenarios [13,14,15]. However, one major difficulty when designing training tasks is in adjusting the level of difficulty/complexity [40] and promoting the effective transfer to the competitive context. In this study, training tasks with lower DM (i.e., Low and Mod DM) led to a higher frequency of actions compared to more complex and representative tasks (i.e., High DM). In contrast, the High DM tasks were the only ones that promoted more clear improvements in the players’ performance. Therefore, while some tasks seem to stress the frequency of actions under contexts of lower contextual information (e.g., Low DM), neglecting the positioning on the field to ensure the perception and action cycle, in turn, more representative tasks with higher complexity and DM requirements may decrease the number of actions but guide and stress the emergence of such actions according to the requirements of a competitive environment [13,52]. However, such an idea cannot be generalised, and coaches need to understand the impact of each manipulation on the different factors that sustain players’ performance. Accordingly, the results of the Mod DM tasks showed a decrease in the players’ ability to identify teammates in a more offensive position. Considering the task design, this finding may be expected; that is, the Mod DM task consisted of a task where the players were static in the square and, when in possession, would have to pass the ball towards one teammate on the right, the left, or in front. In other words, the player had to pass the ball to one of two options, as the third option was expected to have the second ball. For instance, while the player must scan for the body orientation of the player in possession of the second ball to anticipate their passing direction, or for the ball’s trajectory to avoid hitting it, the passing solutions are in a static position. Thus, in this task, the players had no intention of progressing on the field and conquering space, as result of it being a static ball-passing task. Despite not revealing significant differences, decrements were also identified following the Low DM task, in which players were told how to control the ball at each moment. In contrast, players showed higher mean values of ball control skills after the High-DM task, possibly because the players had to adapt their ability to control the ball each time according to their teammates’ and opponents’ positions in order to be successful. Additionally, improvements were also found for the passing DM and execution after the High DM task.

These results may shed some light on the impact of the task design on the subsequent performance, as the manipulation of each task clearly changes the positioning of the players and, consequently, the perceptual–motor landscape and the intentional exploration of possibilities for action [13,52]. In the long-term, such exposure to training tasks that highlight the relevant information for DM in game situations is likely to help players to become attuned with their environment [53]. Altogether, these results suggest that coaches should consider designing tasks that encourage players’ to improve their positioning so as to identify and sustain their actions based on the relevant information from the environment [5,54,55]. For this purpose, practice tasks must highlight the process that promotes the co-dependence between the performer and the surrounding environment [41], which can be achieved by using game-based tasks [13] or tasks with reduced variability in the context of play but that promote the information–action coupling required to perform successfully. Such situations expose the players to a guided intentional exploration of the context of play as required in the context of performance [36] and, ultimately, may enhance their performance in competitive settings. 

### 4.2. Comparing the Interventions’ Acute Effects between Groups (Between-Group Analysis)

One strategy often adopted by coaches to model the training tasks according to the players’ level is to adjust their difficulty and complexity [56] by varying the number of available opportunities for action and, consequently, the DM process [15,54]. For this purpose, coaches may change the boundary conditions during SSGs. For example, Práxedes, Moreno, Gil-Arias, Claver, and Del Villar [36], Machado, Barreira, Teoldo, Travassos, Júnior, Santos, and Scaglia [56], and Pizarro et al. [57] revealed improvements in performances in passing DM and execution in the low-difficulty/DM scenarios (i.e., numerically unbalanced tasks or during SSGs with a lower number of players). Apart from variations in the number of players during SSGs, coaches often vary the level of task DM by adopting more prescriptive and repetitive passes or tasks without opposition [7,40]. In the present study, the level of DM was manipulated by adopting prescriptive and repetitive tasks (i.e., Low DM), without opposition, but with variability and contextual dependence (i.e., Moderate DM) and game-based tasks (i.e., High DM).

When comparing the pre- and post-test interventions between groups, the results showed effects mostly for the ball control variables and between the Low DM and Mod DM tasks. Previous studies comparing repetitive approaches with tasks grounded by variability have found higher brain activation during less predictable practices [58]. Such differences are related to the alpha and theta waves, which are related to the somatosensory information (such as motor, visual, or proprioceptive sensory integration) [59] and motor performance [60]. In contrast, it is expected that players may disengage when exposed to periods of repetitive practice [61], which may affect subsequent practices as well as contributing to performance decrements following the post-test. Additionally, differences were also found between the Low DM and Mod DM tasks for sprinting. In this respect, since Mod DM decreased the ability to find a player in a more offensive position following the intervention, the tactic of sprinting towards the opponents’ goal may have emerged as a suitable strategy to progress. In fact, sprinting is the most common action performed prior to a goal [62], which may reinforce the current findings. Additionally, effects were also found between the Low DM and High DM tasks for appropriateness, which refers to the players’ ability to receive the ball with the relevant body part (e.g., using the furthest foot, as it allows control of the ball towards the space), with higher values for the High DM group. Following the same rationale, the players were constantly challenged to adapt the way they received the ball during the High DM intervention; that is, while at the first ball reception the player may have numerical superiority, conferring higher space and tempo to receive the ball, during the second one they may have two defenders close, affecting the way in which they must adapt their ball reception, while they may use their chest to receive a high pass during the third ball reception. Altogether, the unpredictability that emerges from a game-based task also seems to stress the players’ ability to receive the ball in more suitable conditions to progress. 

Overall, these findings have important practical implications for coaches. Despite being aware of the importance of game-based situations, only a limited amount of time is spent with these types of practices [63]. Thus, coaches may reflect on the nature of their practices and the corresponding consequences for their players’ learning. This exploratory study intends to provide additional information to assist coaches in designing training tasks. Accordingly, tasks with lower DM seem to stress the frequency of motor skills. However, the lower contextual dependency and DM seem to limit the transfer to subsequent performance. In contrast, game-based situations are likely to help players couple their actions with the relevant information [13], contributing to improvements in their ability to control and pass the ball. However, each manipulation should be analysed to further understand the intentions that the tasks promote in the players’ positioning, DM, and action. 

Despite the important practical applications derived from the present study, some limitations should be acknowledged. Firstly, and most importantly, the low sample size may prevent us from achieving stronger inferences. Moreover, players with different levels of ability seem to adapt differently to similar game conditions; thus, a more comprehensive understanding might emerge if further studies consider samples of different ages, ability levels, and genders. Additionally, this study adopted an approach in which the intervention lasted for 6 min, which could explain the small effects identified. The extent to which shorter or longer periods might contribute to increased or reduced effects is still unknown and, thus, should be considered by researchers and practitioners. Lastly, one major aim when planning and designing training sessions is to apply tasks that can be transferred to the subsequent game. For example, Práxedes et al. [64] explored how a previous task (5 vs. 5 ball possession in numerical equality or 5 vs. 4 ball possession in numerical superiority) affected players’ ability to maintain possession during a subsequent 5 vs. 5 game, finding better results when the players were first given numerical superiority. Therefore, further research is required to better understand how to tailor the training session sequence to optimise the learning environment. 

## 5. Conclusions

The results of this study support a current series of literature suggesting the need for more research that explores motor skill acquisition in team sports. Often, coaches use prescriptive tasks in different stages of performance, such as in youth football to refine technical skills, or at elite levels during congested fixtures to develop specific movement patterns due to the Low DM and external load requirements (e.g., exploring specific passing sequences that were identified as weaknesses during the opposition analysis). Training tasks grounded by low-to-moderate DM can be used to increase the frequency of technical actions. However, as hypothesised, such tasks have lower performance transfer compared to game-based situations—mainly in terms of ball-control-related variables and distance covered while sprinting (Low DM), and in the ability to find players in more offensive positions (Mod DM). These types of tasks have predetermined movement sequences (Low DM) or are performed without opposition (Mod DM) and, thus, do not consider the dynamic cooperation and opposition interactions that characterise competitive performance. In contrast, the High DM task was designed according to those principles, emphasising players’ ability to receive the ball oriented to the space. Moreover, as hypothesised, the results from this study showed that more static tasks might limit the players’ ability to identify teammates in more offensive positions. Thus, considering the tight time schedules for practice and priorities for development, coaches may carefully consider what practices should be adopted to enhance players’ performance. 

## Figures and Tables

**Table 1:** Descriptive (M ± DP) statistics from the intervention tasks (Low DM, Mod DM, and High DM).

Task Performance	Low Decision	Moderate Decision	High Decision	Difference in Means (% ± 95% CI)		
	(Mean ± SD)	(Mean ± SD)	(Mean ± SD)	Low DM vs. Mod DM	Low DM vs. High DM	Mod DM vs. High DM
Ball Control						
Unsuccessful Ball Control (n/6 min)	0.13 ± 0.16	0.21 ± 0.16	0.06 ± 0.09	0.0 ± 0.0	−0.33 ± 1.43	−0.3 ± 1.43
Successful Ball Control (n/6 min) (n/6 min)	3.54 ± 0.28	15.58 ± 2.17	1.92 ± 1.07	72.25 ± 10.71	−9.75 ± 6.56	−82.0 ± 8.00
Pass Behaviour						
Unsuccessful Pass (n/6 min)	0.25 ± 0.22	0.79 ± 0.63	0.46 ± 0.29	3.25 ± 2.56	1.25 ± 1.40	−2.00 ± 3.46
Successful Pass (n/6 min)	3.63 ± 0.25	16.92 ± 1.97	1.90 ± 0.93	79.75 ± 10.04	−10.38 ± 5.47	−90.13 ± 0.87

Note: N = number; CI = confidence interval.

**Table 2:** Descriptive (M ± DP; Me ± IQR; Raw ± 95% CI) and inferential statistics from the passing intervention according to the conditions (Low DM, Mod DM, and High DM).

	**Low DM**		**Moderate DM**		**High DM**		**Difference in Means (Raw ± 95% CI)**			***p***
**Motor Execution and DM**	**Pre-Test**	**Post-Test**	**Pre-Test**	**Post-Test**	**Pre-Test**	**Post-Test**				
	**(Mean ± SD)** **[Median ± IQR]**	**(Mean ± SD)** **[Median ± IQR]**	**(Mean ± SD)** **[Median ± IQR]**	**(Mean ± SD)** **[Median ± IQR]**	**(Mean ± SD)** **[Median ± IQR]**	**(Mean ± SD)** **[Median ± IQR]**	**Low DM vs. Mod DM**	**Low DM vs. High DM**	**Mod DM vs. High DM**	
**Ball Control (GPET) and Criteria**										
Execution	[0.81 ± 0.37]	[0.78 ± 0.37]	[1.00 ± 0.00]	[1.00 ± 0.00]	[0.89 ± 0.25]	[0.86 ± 0.20]	0.06 ± 0.18	0.10 ± 0.21	0.04 ± 0.15	**0.032 ^a^**
Appropriateness	[1.00 ± 0.00]	[1.00 ± 0.00]	[1.00 ± 0.00]	[1.00 ± 0.00]	[1.00 ± 0.00]	[1.00 ± 0.00]	0.18 ± 0.23	0.11 ± 0.22	−0.07 ± 0.13	**0.017 ^a,b^**
Body Orientation	(0.38 ± 0.25)	[0.50 ± 0.57]	(0.55 ± 0.28)	(0.64 ± 0.18)	(0.50 ± 0.26)	(0.66 ± 0.19) **^#^**	−0.02 ± 0.30	0.05 ± 0.30	0.07 ± 0.20	0.167
Motor Space	[0.50 ± 0.57]	[0.50 ± 0.57]	[0.50 ± 0.57]	[0.50 ± 0.57]	[0.50 ± 0.57]	[0.50 ± 0.57]	0.07 ± 0.18	0.1 ± 0.21	0.03 ± 0.16	**0.028 ^a^**
**Pass (GPET) and Criteria**										
Execution	(0.88 ± 0.13)	[0.91 ± 0.34]	(0.86 ± 0.19)	[0.96 ± 0.20]	(0.87 ± 0.12)	[0.96 ± 0.20]	−0.04 ± 0.16	0.01 ± 0.12	0.01 ± 0.12	0.523
Decision-Making	[0.79 ± 0.35]	[0.80 ± 0.37]	[0.77 ± 0.29]	[0.85 ± 0.28]	(0.83 ± 0.31)	[0.84 ± 0.31]	0.09 ± 0.18	0.03 ± 0.22	−0.06 ± 0.19	0.512
Freedom from Opposition	[1.00 ± 0.21]	[0.91 ± 0.23]	[0.89 ± 0.22]	[1.00 ± 0.11]	(0.88 ± 0.14)	[1.00 ± 0.19]	0.16 ± 0.19	0.00 ± 0.20	−0.10 ± 0.12	0.291
Offensive Position	(0.79 ± 0.19)	[0.86 ± 0.38]	[0.82 ± 0.34]	[0.75 ± 0.14] **^#^**	(0.83 ± 0.14)	[0.87 ± 0.30]	−0.05 ± 0.21	−0.05 ± 0.19	0.04 ± 0.14	0.589
Rhythm and Tempo	[0.87 ± 0.30]	[0.87 ± 0.30]	(0.75 ± 0.20)	[0.85 ± 0.31]	(0.75 ± 0.20)	(0.77 ± 0.19)	0.04 ± 0.20	−0.04 ± 0.21	−0.08 ± 0.17	0.416
**Physical Performance**										
Total Distance Covered (m)	(599.09 ± 62.63)	(591.17 ± 44.46)	(587.11 ± 75.41)	(572.67 ± 63.56)	(571.29 ± 55.67)	(561.59 ± 78.6)	−6.53 ± 38.93	−1.79 ± 40.77	4.74 ± 45.16	0.741
Distance Covered While Walking (m)	(68.16 ± 15.61)	(68.81 ± 8.12)	(76.81 ± 19.36)	(80.65 ± 15.67)	(74.97 ± 11.80)	(79.58 ± 22.89)	3.18 ± 11.66	3.95 ± 12.33	0.77 ± 13.80	0.270
Distance Covered While Jogging (m)	(490.38 ± 61.88)	(475.31 ± 51.09)	(471.27 ± 72.09)	(447.32 ± 60.16)	(464.1 ± 51.89)	(457.23 ± 83.79)	−8.88 ± 45.18	8.20 ± 43.56	17.08 ± 51.99	0.658
Distance Covered While Running (m)	[29.8 ± 26.6]	(45.26 ± 24.05)	(36.33 ± 26.35)	(38.22 ± 23.02)	(31.47 ± 19.14)	(24.44 ± 22.08)	−5.58 ± 18.63	−14.50 ± 20.69	−8.92 ± 18.83	0.078
Distance Covered While Sprinting (m)	[29.8 ± 26.6]	[29.8 ± 26.6]	[29.8 ± 26.6]	[29.8 ± 26.6]	[29.8 ± 26.6]	[29.8 ± 26.6]	4.75 ± 4.71	0.56 ± 2.86	−4.20 ± 4.30	**0.042 ^a^**
Average Speed (m/s)	(21.40 ± 2.24)	(21.11 ± 1.57)	(20.99 ± 2.63)	(20.63 ± 2.23)	(17.99 ± 7.22)	(17.54 ± 7.37)	−0.07 ± 1.38	−0.22 ± 1.58	−0.08 ± 1.54	0.715

Note: m = metres; m/ s = metres per second; DM = decision-making; CI = confidence interval. Due to the presence of normal and non-normal data, descriptive data are presented as the mean ± standard deviation (SD) and expressed using (), while the non-normal data are presented as the median ± interquartile range (IQR) and expressed using []. For example, motor execution of ball control for the Low DM task [0.81 ± 0.37] consisted of non-normal data, while body orientation (0.38 ± 0.25) consisted of normal data. Differences between the pre- and post-test design measurements (within analysis) are identified with the # symbol in the descriptive data columns (i.e., # in body orientation for the High DM means statistically significant differences from the pre-test to the post-test). Additionally, differences between the pre-and post-tests of the different interventions are identified by bold values, while letters represent statically significant differences between groups (between-group comparison) based on the differences in the pre- and post-test measurements: (i) Low DM vs. Mod DM, (ii) Low DM vs. High DM, and (iii) Mod DM vs. High DM.

## Data Availability

In order to protect the subjects’ confidentiality and privacy, data are only available upon request. Interested researchers may contact the board from the Research Center in Sports Sciences, Health Sciences, and Human Development to request access to the data (cidesd.geral@utad.pt).
